# The effect of high-top and low-top shoes on ankle inversion kinematics and muscle activation in landing on a tilted surface

**DOI:** 10.1186/1757-1146-7-14

**Published:** 2014-02-18

**Authors:** Weijie Fu, Ying Fang, Yu Liu, Jianfu Hou

**Affiliations:** 1 Key Laboratory of Exercise and Health Sciences of Ministry of Education, Shanghai University of Sport, Shanghai 200438, China; 2 Department of Kinesiology, Recreation, and Sport Studies, The University of Tennessee, Knoxville 37996, TN, USA

**Keywords:** High-top/low-top shoe, Ankle inversion, Muscle pre-activity, Tilted surface, Landing

## Abstract

**Background:**

There is still uncertainty concerning the beneficial effects of shoe collar height for ankle sprain prevention and very few data are available in the literature regarding the effect of high-top and low-top shoes on muscle responses during landing. The purpose of this study was to quantify the effect of high-top and low-top shoes on ankle inversion kinematics and pre-landing EMG activation of ankle evertor muscles during landing on a tilted surface.

**Methods:**

Thirteen physical education students landed on four types of surfaces wearing either high-top shoes (HS) or low-top shoes (LS). The four conditions were 15° inversion, 30° inversion, combined 25° inversion + 10° plantar flexion, and combined 25° inversion + 20° plantar flexion. Ankle inversion kinematics and EMG data of the tibialis anterior (TA), peroneus longus (PL), and peroneus brevis (PB) muscles were measured simultaneously. A 2 × 4 (shoe × surface) repeated measures ANOVA was performed to examine the effect of shoe and landing surfaces on ankle inversion and EMG responses.

**Results:**

No significant differences were observed between the various types of shoes in the maximum ankle inversion angle, the ankle inversion range of motion, and the maximum ankle inversion angular velocity after foot contact for all conditions. However, the onset time of TA and PB muscles was significantly later wearing HS compared to LS for the 15° inversion condition. Meanwhile, the mean amplitude of the integrated EMG from the 50 ms prior to contact (aEMG_pre_) of TA was significantly lower with HS compared to LS for the 15° inversion condition and the combined 25° inversion + 20° plantarflexion condition. Similarly, the aEMG_pre_ when wearing HS compared to LS also showed a 37.2% decrease in PL and a 31.0% decrease in PB for the combined 25° inversion + 20° plantarflexion condition and the 15° inversion condition, respectively.

**Conclusion:**

These findings provide preliminary evidence suggesting that wearing high-top shoes can, in certain conditions, induce a delayed pre-activation timing and decreased amplitude of evertor muscle activity, and may therefore have a detrimental effect on establishing and maintaining functional ankle joint stability.

## Background

Ankle sprain is one of the most common injuries in basketball athletes, with reported incidence rates of 3.85 per 1000 participations [1] and 5.5 injuries per 1000 activity hours [2]. These lateral ligament sprains usually occur during touchdown with excessive inversion and plantarflexion of the foot when athletes land on an uneven surface, or perform a lateral cutting maneuver [3]. Hence several preventive measures have been suggested over the past 40 years which seek to change the ankle landing kinematics and thus decrease the occurrence of ankle sprains by using specially designed high-top shoes or other external support [4-6].

The function of high-top shoes in preventing ankle sprains has been widely studied since the 1980s [7]. However, no scientific consensus has been reached yet with regard to the stabilizing effect of high-top shoes in restricting ankle inversion. A number of studies have reported that high-top shoes in comparison to low-top shoes decreased the amount and rate of inversion, and further decreased the risk of ankle sprains [8,9]. The possible biomechanical reasons for this have been mainly attributed to limiting ankle inversion ROM [7] and decreasing external joint stress [10]. In contrast, from an epidemiological viewpoint, no significant differences were found in ankle sprain occurrence between high-top shoes (4.80 × 10^-4^ injuries per player-minute) and low-top shoes (4.06 × 10^-4^ injuries per player-minute) [11,12]. Rovere *et al.*[13] reported that high-top shoes were not more effective than low top shoes, and the fewest injuries were observed with low top shoes and “laced ankle stabilizers”. Similarly, high-top shoes did not show superiority over low-top shoes in preventing ankle sprains especially for those who had no history of ankle sprains [14]. Therefore, there is uncertainty concerning the beneficial effects of shoe design for ankle sprain prevention and very few data are available in the literature regarding the effect of shoe collar height on muscle responses, which further hinders our understanding of the potential mechanisms underlying shoe effects.

The pre-landing muscle activity of the lower leg, typically of the peroneus longus (PL), peroneus brevis (PB), and tibialis anterior (TA), has been investigated previously [15-17]. Such muscle activity, which is also termed pre-activation or preparatory muscle activity, has been found to contribute to restricting the ankle from plantarflexion and inversion in many instances of ankle sprains [16,18]. Theoretically, earlier onsets and larger amplitudes of pre-landing EMG activity are considered crucial to prepare the foot positioning at touch-down and throughout the subsequent joint movements [19]. Specifically, the EMG pre-activation can stiffen the muscle in preparation for the landing and continues through the contact [20]. An appropriate level of initial muscle stiffness enables the elastic energy to be stored and released from the muscle - tendon complex [21] and limits joint rotations after foot contact [22]. The failure of muscle to control joint ROM, which greatly relies on muscle pre-activity prior to landing, may lead to injuries [23]. Consequently, sufficient pre-landing EMG activity is considered to be a mechanism acting to protect the ligaments and joints from fall-related injury [24]. Since various shoe designs (differing in aspects such as cushioning properties and ankle collar characteristics) may provide different proprioceptive inputs, different neuromuscular responses of the lower leg muscles may be exhibited correspondingly during the fall [10]. Thus, it seems logical to assume that high-top and low-top shoes will have different influences on preparatory muscle activity during landing, which may, in turn, provide a better understanding of the collar height effect.

The vast majority of studies to date attempting to replicate the inversion movement that occurs during a lateral ankle sprain have utilized trapdoor devices [9,17,25,26]. While this approach has provided useful information about the development of lateral ankle sprains and the stabilizing effect of high-top shoes, the validity of these devices in replicating the mechanism of ankle sprain has been questioned as, in real life, sprains are not typically caused by trapdoor falls [27,28]. Furthermore, a recent study suggests that earlier maximum inversion angles and greater inversion velocities are produced during inverted surface landing compared with the traditional inversion drop movement on a trapdoor [29]. Such a type of surface landing has recently been shown to be more demanding and worthy of consideration for investigations of ankle support and lateral ankle performance/injury mechanisms [29,30]. However, to our knowledge, landing onto an inverted surface has rarely been used in testing the stabilizing effect of high-top shoes in restricting ankle inversion.

Based on the above observations, the purpose of this study was to examine the effect of high-top and low-top shoes on ankle inversion kinematics (maximum ankle inversion and inversion velocity) and pre-landing EMG activation of ankle evertor muscles during landing on a tilted surface. We hypothesized that a high-top shoe intervention would decrease ankle inversion and increase EMG pre-activation during inverted landings.

## Methods

### Participants

Thirteen healthy male physical education students (age: 21.3 ± 1.2 years, height: 178.6 ± 3.8 cm, weight: 69.9 ± 5.9 kg) volunteered to participate in this study. All of them were free of musculoskeletal injuries of the lower extremity within the past six months, and were instructed to refrain from strenuous exercise during the 24 hours preceding the tests. Each participant signed an informed consent form approved by the Ethics Committee of Shanghai University of Sport prior to experimental testing. A post-hoc power analysis was executed to indicate the statistical power. It revealed that a sample size of 13 was sufficient to minimize the probability of Type II error for our variables of interest.

### Testing shoes

Customized high-top and low-top basketball shoes from the same footwear manufacturer were used in this study. The two types of prototypes had identical outsole and midsole. The only difference was a 6 cm difference in shoe collar height, which was taken from the very top of each shoe. Meanwhile, the participants were instructed to pull the laces tight, beginning with the bottom set of eyelets and moving all the way upward. Additionally, socks were worn to avoid relative movement between shoes and foot.

### Tilted platform

**Figure 1:**
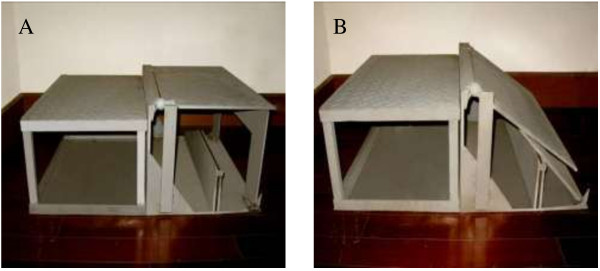
**Tilted platform. (A)** flat and **(B)** 30° inversion (could also initiate 15° inversion, 25° inversion + 10° plantarflexion, and 25° inversion + 20° plantarflexion).

A tilted platform [72 cm (L) × 63 cm (W) × 30 cm (H)] which could induce ankle inversion and/or plantarflexion was used in this study (Figure 1). Specifically, the customized platform was used to tilt to 1) 15° inversion, 2) 30° inversion, 3) combined 25° inversion and 10° plantarflexion, and 4) combined 25° inversion and 20° plantarflexion. The platform was tilted and fixed before the landing. The participants landed on the tilted platform with the dominant foot and the flat platform with the other foot. The tilted plate was 5 cm higher than the flat plate in order to guarantee that the participant made contact with the inverted surface with the dominant foot first [29]. A tilt combining inversion and plantarflexion was selected because ankle sprains often occur in this position [31]. We also chose to limit the amount of inversion to 30° in order to remain within the safe range of foot/shoe motion, as injury to the lateral ankle ligaments may occur when the ankle exceeds 40° of inversion [26,32].

### Testing protocol

**Figure 2:**
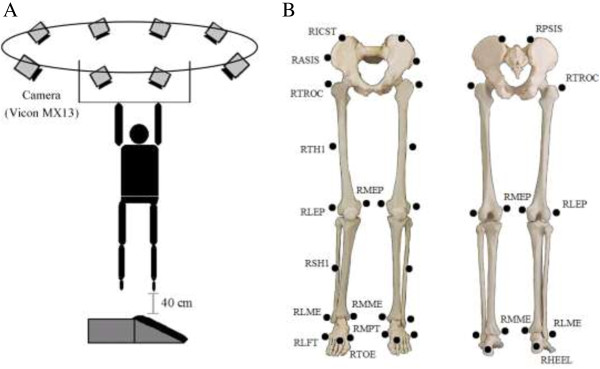
**Experimental setup (A) and reflective marker set used in the present study (B).** The following describes in detail where the markers were placed on the foot. Where right side markers only are listed, the positioning is identical for the left side. RLEP, RMEP: placed on the lateral and medial epicondyle of the knee; RSH1: a tracking marker on the shank; RLME, RMME: placed on the lateral and medial malleolus along an imaginary line that passes through the transmalleolar axis; RHEEL, RTOE: placed on the calcaneous and the second metatarsal head; RLPT, RMPT: placed over the fifth and first metatarsal head.

The participants were instructed to hang from an overhead bar with their heels 40 cm from the platform (Figure 2A). They were required to keep their eyes straight ahead during the entire procedure. The inversion (or plantarflexion) angle of the tilted platform was then adjusted and determined by an experimenter. After that the participants were asked to perform a self-initiated drop landing. The platform did not move when the participant landed on it. It was tilted and fixed in the chosen configuration prior to landing. The exact degree of inclination of the platform was unknown to the participants during the entire testing procedure in order to eliminate partial effects of pre-knowledge. The order of the platform tilt conditions as well as the shoes was randomized. Participants were given sufficient practice trials to be able to familiarize themselves with the drop landing task. After a regular warm-up routine and practice trials, the formal testing began. Five successful trials in each condition, i.e. 15° inversion, 30° inversion, 25° inversion + 10° plantarflexion, and 25° inversion + 20° plantarflexion, were required. A rest period of two minutes was provided between trials. A successful trial was defined as one in which the participant adopted a stable landing posture (without losing their balance) on the surface at the end of the landing.

### Kinematics

An 8-camera motion analysis system (Vicon MX series, camera MX13, Oxford Metrics, UK) was used to obtain the frontal plane kinematics of the dominant lower extremity at a sampling rate of 120 Hz. 28 retroreflective markers (14.0 mm diameter) comprising the plug-in gait marker set were attached to both lower limbs to define hip, knee, and ankle joints [33] (Figure 2B). Specifically, reflective markers were placed on the following locations to define the ankle joint: lateral and medial epicondyle of the knee, the shank (for a tracking marker), lateral and medial malleoli of the ankle, the first and fifth metatarsal heads, the second metatarsal heads and calcaneous. The ankle joint center was defined as the midpoint between the medial and lateral aspects of the malleoli markers. The 3D coordinates of all reflective markers were filtered through a Butterworth fourth-order, zero-lag, low-pass filter at a cut-off frequency of 7 Hz [24]. Kinematic variables of interest included: 1) the ankle inversion angle at contact (*θ*_cont_); 2) the maximum ankle inversion angle (*θ*_max_); 3) the time to maximum ankle inversion angle (*θ*_t-max_); 4) the ankle inversion range of motion (ROM); 5) the maximum ankle inversion angular velocity after foot contact (*ω*_max_); 6) the time to the maximum ankle inversion angular velocity (*ω*_t-max_); 7) the average ankle inversion angular velocity (determined by dividing the amount of inversion by the time to maximum inversion after initial foot contact, *ω*_ave_). The timing of the foot contact was determined by the force plate. Two 90 × 60 cm force plates (9287B, Kistler Corporation, Switzerland) fixed beneath the tilted platform were employed to capture ground reaction force data at a sampling rate of 1200 Hz. The ground reaction force and kinematic data were sampled simultaneously using the Vicon system. All the above variables were calculated using Visual 3D software (4.00.20, C-Motion Inc., U.S.A.) [34]. In the current study, Visual 3D calculated joint angles using a Cardan sequence of rotations. The Cardan sequence for the calculation of joint angles is x-y-z, which is equivalent to flexion/extension – abduction (inversion)/adduction (eversion) – axial rotation.

### Electromyography

A 16-channel Biovision system (Biovision, Wehrheim, Germany) was simultaneously used to record the EMG from the tibialis anterior (TA), peroneus longus (PL), and peroneus brevis (PB) muscles of the dominant leg at a sampling frequency of 1200 Hz (input impedance = 10^12^ Ω, common-mode rejection ratio = 120 dB at 60 Hz). Prior to the placement of EMG electrodes, the skin of the participant was carefully prepared (shaved, abraded with sandpaper, and cleaned with rubbing alcohol) to reduce skin impedance [35]. Disposable bipolar Ag/AgCl surface electrodes were placed on the referenced positions of these muscles [26,27]. For the PL muscle, the electrode was placed at the junction of the proximal and middle thirds of the fibula over the palpable lateral compartment. The electrode for the PB muscle was placed three quarters of the distance between the fibular head and the lateral malleolus, immediately anterior to the PL tendon. For the TA muscle, the electrode was placed at the junction of the proximal and middle thirds of the tibia, over the largest portion of the muscle belly. Proper electrode placement was verified by observing the EMG signal on a computer monitor during maximum voluntary ankle eversion and plantar flexion to ensure that there was no crosstalk present from adjacent muscles [18]. The same experimenter completed the procedures for each participant to control for differences in preparation and placement techniques.

The EMG data were analyzed using DASYLab software (8.0, DATALOG GmbH, Moenchengladbach, Germany). The raw signals were band-pass filtered at 10 – 500 Hz, and then full-wave rectified [36]. The variables of interest included: 1) The onset time of pre-landing EMG activity (the time when muscle contractions were initiated before foot contact); 2) The mean amplitude of the integrated EMG from the 50 ms prior to initial contact (aEMG_pre_) [19], which was calculated using the following equations:

$$\mathrm{IEMG}=\int_{t}^{t+T}\left|\mathrm{EMG}\left(t\right)\right|\cdot \mathrm{dt}$$

$$\mathrm{Mean}\;\mathrm{Amplitude}=\frac{1}{T}\mathrm{IEMG}$$

Where *t* is the onset of signal and *T* is the time interval.

**Figure 3:**
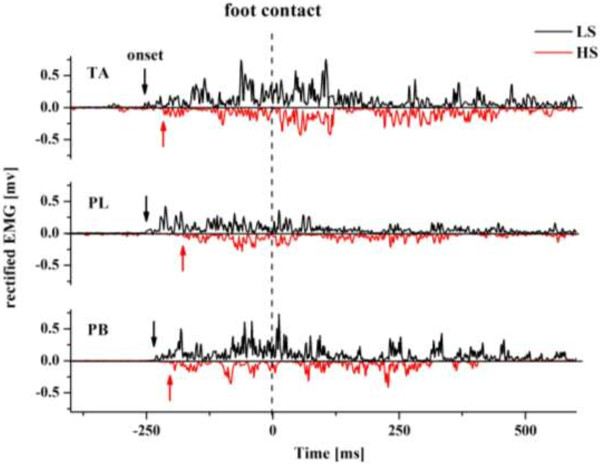
**Full-wave rectified EMG curves during landing on a 15° inversion surface.** Representative full-wave rectified EMG curves of the tibialis anterior (TA), peroneus longus (PL), and peroneus brevis (PB) muscles during landing on a 15° inversion surface wearing high-top (HS) and low-top shoes (LS). Arrows indicate the onset time of pre-landing EMG activity (0 ms was defined as the time of foot contact). HS data inverted to allow both curves to be visualized.

Figure 3 shows representative full-wave rectified EMG curves of the TA, PL and PB muscles during landing on a 15° inversion surface in wearing two types of shoes. The arrows indicated the onset time of the TA, PL and PB, which were determined via visual inspection. Specifically, EMG onset was defined on the basis of the earliest detectable rise in activity beyond the steady state level of activation [37,38]. In most cases this measure was made easy by the absence of detectable background activity in the muscles being recorded (Figure 3). In order to reduce observer bias, the inspection was performed by the same experienced investigator who was blind to the shoe and tilt condition [39].

### Statistical analysis

The distribution of all dependent variables was examined by using the Shapiro-Wilk test and was found not to differ significantly from normality. A 2 × 4 (shoe × surface) repeated measures analysis of variance was performed to examine the effect of shoe and landing surfaces on ankle inversion and EMG responses. Tukey post hoc tests were performed when significant main effect or interaction was observed (16.0, SPSS Inc., Chicago, IL, U.S.A.). The significance level for all tests was set at α=0.05.

## Results

### Ankle inversion kinematics

A main effect of landing surfaces was observed on the ankle inversion kinematics, but not the shoe type; there was no significant shoe × surface interaction among the ankle inversion kinematical variables. No significant differences in the maximum ankle inversion angle (*θ*_max_), the ankle inversion range of motion (ROM), and the maximum ankle inversion angular velocity after foot contact (*ω*_max_) were found between wearing high-top shoes and low-top shoes for all conditions (Table 1). Specifically, the values of *θ*_max_, ROM, *ω*_max_, and *ω*_ave_ generally increased with increased surface inversion angle from 15° to 30° (or 25°) (Table 1).

**Table 1:** Comparison of ankle inversion kinematic variables (mean ± SD) in wearing high-top (HS) and low-top shoes (LS) in four surface conditions

**Variables**	**Shoe condition**	**Surface condition**			
		**15°_Inv**	**30°_Inv**	**25°_Inv + 10°_PF**	**25°_Inv + 20°_PF**
*θ*_cont_ (°)	HS	11.8 ± 5.1	13.2 ± 4.6	13.7 ± 5.3	12.4 ± 5.3
	LS	12.4 ± 4.3	12.4 ± 5.3	12.2 ± 4.8	11.7 ± 5.5
*θ*_max_ (°)	HS	14.8 ± 6.3	28.3 ± 6.7^*^	23.6 ± 3.4^*^	23.9 ± 5.4^*^
	LS	15.2 ± 5.3	29.3 ± 4.7^*^	25.0 ± 5.1^*^	25.7 ± 6.5^*^
*θ*_t-max_ (ms)	HS	48.3 ± 21.4	67.2 ± 43.2	56.0 ± 33.8	62.2 ± 34.1
	LS	44.6 ± 26.8	76.1 ± 29.7	45.4 ± 39.9	55.2 ± 27.5
ROM (°)	HS	2.4 ± 0.9	15.2 ± 6.1^*^	12.1 ± 4.4^*^	13.0 ± 6.1^*^
	LS	2.7 ± 1.1	17.4 ± 5.7^*^	13.8 ± 5.5^*^	14.4 ± 3.7^*^
*ω*_max_ (°/s)	HS	62.9 ± 32.5	208.7 ± 112.9^*^	189.0 ± 109.0^*^	194.6 ± 106.2^*^
	LS	61.5 ± 29.3	220.2 ± 125.6^*^	201.6 ± 115.2^*^	232.5 ± 127.0^*^
*ω*_t-max_ (ms)	HS	21.4 ± 12.5	33.7 ± 24.8	36.9 ± 22.3	34.0 ± 24.3
	LS	24.3 ± 14.1	30.1 ± 20.4	32.1 ± 27.7	25.3 ± 18.4^*^
*ω*_ave_ (°/s)	HS	49.2 ± 12.5	150.6 ± 60.2^*^	142.8 ± 52.4^*^	148.6 ± 64.5^*^
	LS	46.6 ± 16.3	151.3 ± 76.9^*^	150.2 ± 69.9^*^	158.3 ± 77.6^*^

*θ*_cont_, ankle inversion angle at contact; *θ*_max_, maximum ankle inversion angle; *θ*_t-max_, time to the maximum ankle inversion angle; ROM, ankle inversion range of motion; *ω*_max_, maximum ankle inversion angular velocity after foot contact; *ω*_t-max_, time to the maximum ankle inversion angular velocity; *ω*_ave_, average ankle inversion angular velocity; 15°_Inv, 15° inversion; 30°_Inv, 30° inversion; 25°_Inv + 10°_PF, combined 25° inversion and 10° plantarflexion; 25°_Inv + 20°_PF, combined 25° inversion and 20° plantarflexion.

^*^ significantly different from 15°_Inv in the same shoe condition (*p* < 0.05).

### Pre-landing EMG activity

**Figure 4:**
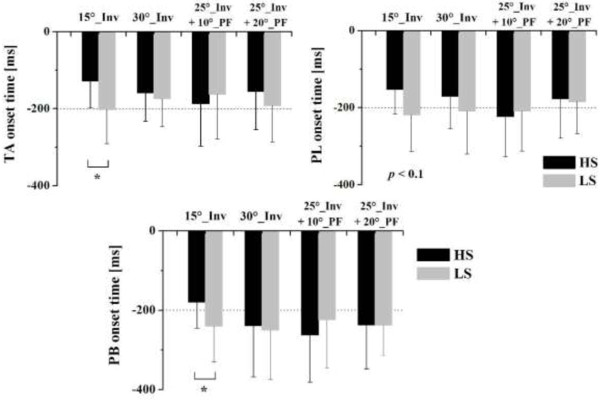
**Comparison of onset time between the two shoe conditions.** Comparison of the onset time of the tibialis anterior (TA), peroneus longus (PL), and peroneus brevis (PB) muscles between wearing high-top (HS) and low-top shoes (LS) in four surface conditions. ^*^indicates significant differences between HS and LS (*p* < 0.05).

A main effect of shoe types was observed on the onset time and amplitude of pre-landing muscle activity; there was no significant shoe × surface interaction among any outcome variables. The post hoc comparisons showed that the onset time of TA (*F* = 4.486, *p* = 0.047) and PB (*F* = 4.476, *p* = 0.048) muscles was significantly later when wearing high-top shoes compared to low-top shoes for the 15° inversion condition (Figures 3 and 4). In addition, wearing high-top shoes also delayed the onset time of PL (*F* = 3.238, *p* = 0.089). However, for the other three surface conditions, i.e. 30° inversion, combined of 25° inversion + 10° plantar flexion, and combined of 25° inversion + 20° plantarflexion, no significant differences of the onset time of the TA, PL and PB were found between wearing high-top shoes and low-top shoes.

**Figure 5:**
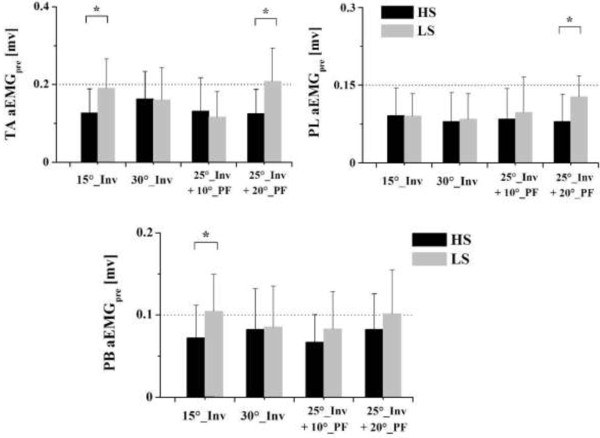
**Comparison of mean EMG amplitude (aEMGpre) between the two shoe conditions.** Comparison of the aEMGpre of the tibialis anterior (TA), peroneus longus (PL), and peroneus brevis (PB) muscles between wearing high-top (HS) and low-top shoe (LS) of four surface conditions. ^*^indicates significant differences between wearing HS and LS (*p* < 0.05).

For the amplitude of pre-landing muscle activity, the aEMG_pre_ of TA when wearing high-top shoes was significantly lower compared to low-top shoes while landing in the 15° inversion condition (*F* = 4.727, *p* = 0.035) and the combined 25° inversion + 20° plantarflexion (*F* = 4.782, *p* = 0.033) condition (Figure 5). Similarly, the aEMG_pre_ wearing high-top shoes compared to low-top shoes also showed a 37.2% decrease in PL (*F* = 4.574, *p* = 0.042) and a 31.0% decrease in PB (*F* = 4.539, *p* = 0.046) for the combined 25° inversion + 20° plantarflexion condition and the 15° inversion condition, respectively (Figure 5).

## Discussion

### Ankle inversion

In the current study, we adopted a simulation of a lateral ankle inversion sprain, landings on an inverted (and plantarflexed) platform, to evaluate the influence of high-top and low-top shoes on ankle inversion kinematics [29]. Our results showed no significant shoe effect on the maximum ankle inversion angle, the ankle inversion ROM, and the maximum ankle inversion angular velocity during landing on an inverted or a combined inverted and plantarflexed surface.

Much of the previous work focussing on the stabilizing effect of high-top shoes was conducted during cutting and jumping movements, or using a tilting platform to induce sudden ankle inversion [17,40]. Brizula *et al.*[41] found that the use of high-support shoes resulted in a smaller initial eversion angle and a smaller maximum eversion angle during jump landing. This might be partially ascribed to a forced contact of the sole caused by the increased vertical rigidity of the shoe. On the contrary, during the procedure of sudden ankle inversion induced by a trapdoor, the amount of inversion as well as the maximum rate of inversion in high-top basketball shoes was significantly lower than that in low-top shoes [9]. A possible explanation for these observations is that shoe height may significantly increase the active resistance to an inversion moment and this could reduce the risk of ankle sprains [8]. Collectively, there is disagreement on the effect of high-top shoes in restricting ankle inversion ROM, and few well-designed biomechanical investigations have been conducted to address this issue [4,5]. Our main findings indicated that high-top shoes adopted in this study did not reduce ankle inversion angle, ankle inversion range of motion, and inversion angular velocity compared to low-top shoes after foot contact on a tilted surface. Since no significant differences in inversion kinematics were found between the two shoe conditions, increased height does apparently not provide more “rigidity” as one would intuitively assume. Thus, our results suggest that high-top shoes are not effective in increasing ankle joint stability.

Among the previously published landing studies using high-top shoe, few studies actually used a landing testing protocol on an inverted surface. Therefore, direct comparisons of the results between the prior studies and ours are not appropriate due to the different types of induced ankle inversion movement, as well as the different types of shoes used. From a biomechanical point of view, compared to the inversion induced by a trap door, the inverted surface landing would produce a significantly earlier maximum inversion angle and velocity, and greater inversion velocities [29], which suggests that the inverted surface landing may be more demanding in evaluating ankle inversion performance [30]. In actual sports activities, ankle inversions mostly occur in a dynamic movement and a more plantarflexed ankle positioning [3], such as landing on an irregular surface or on somebody’s foot after jumping, instead of in a static and normal foot position condition [42]. These considerations suggest that a landing onto an inverted surface represents a more realistic simulation of a lateral ankle inversion sprain [29,30]. In the current study, the lack of significant differences in the maximum ankle inversion angle and the maximum ankle inversion angular velocity between the high-top and low-top shoes thus potentially supports the results from the majority of epidemiological studies which have found that no clear differences between high-top and low-top basketball shoes regarding the incidence of ankle sprains [11,12]. However further studies are warranted to investigate more realistic simulation of ankle sprains, and to explore the effect of shoe type on inversion kinematics, imposed inversion stress, and their relevance to sprain occurrences.

### Pre-landing muscle activity

The role of pre-landing EMG activity is important since it prepares the muscle-tendon complex for a rapid, forceful stretch occurring after foot contact and throughout the subsequent joint rotations [19]. In our study, the results showed that the shoe partially influenced the timing and amplitude of evertor muscle activity before touchdown in landing movements. Specifically, a significant later onset time of the tibialis anterior and peroneus brevis muscles was found with high-top shoes compared to low-top shoes for the 15° inversion condition. Meanwhile, the aEMG_pre_ of the tibialis anterior, peroneus longus, and peroneus brevis with high-top shoes was significantly lower compared to low-top shoes while landing under certain conditions (Figure 5).

Despite the fact that the effect of high-top shoes on ankle evertor muscle function has not been systematically investigated previously, there is evidence that shoe characteristics can substantially affect muscle contraction following sudden inversion of the foot [15,17]. Ramanathan *et al.*[17] found the peroneus longus responded earlier in the shod condition compared to barefoot during unanticipated foot inversion. More importantly, among all the shod conditions (standard training shoe, shoe with sole flare, and laced boot), the muscle responded later with the laced boot. These results partially support our findings which also showed a significant later onset time of the TA and PL muscles before contact when wearing high-top shoes. On the other hand, studies focusing on the EMG amplitude found that shod conditions evoked significantly greater muscle contraction following sudden inversion of the foot compared to the barefoot condition [15,17]. It was then speculated that the shoes may have a facilitatory effect and can enhance muscle function [15]. However, in our study, we adopted a landing on an inverted platform rather than using a tilting platform to induce sudden ankle inversion. The aEMG_pre_ of the TA, PL, and PB in participants wearing high-top shoes showed significantly lower levels compared to low-top shoes. This indicates that a smaller muscular effort is required before landing on the inverted surface when wearing high-top shoes. Furthermore, this effect is likely to be dependent on the specific muscle being assessed, the selected shoes, and the landing condition.

It is difficult to explain why there were differences observed in muscle pre-activation between high-top shoes and low-top shoes in some platform tilt configurations but not others, given that participants should, in theory, have been unaware of the tilt of the landing platform. One possible explanation is that participants did in fact have some advance knowledge of the configuration of the landing platform, either by looking down or in some other way, such as other audible or visual clues. As we have stated, the test protocol was designed to prevent this from happening and, whilst it cannot be ruled out completely as a possibility, it is unlikely. A more likely explanation is that the differences identified in the post hoc analyses, which were of marginal statistical significance (0.05 > p > 0.04), were not statistically robust given the relative weakness of the main effect of shoe type.

It remains unclear what direction of change in muscle activity represents a clearly beneficial effect of wearing high-top shoes, and what the intrinsic mechanism of the shoe effect on pre-landing muscle activity is. Since the addition of the shoe was the only extrinsic change implemented during a landing, one of our explanations is that high-top shoes changed proprioceptive input of the foot/ankle complex, which influenced the onset time and magnitude of pre-landing muscle activation. A previous study looking at the effect of bracing on PL activity partially supports this: a delayed PL reaction time after foot contact was found when tight ankle bracing was applied [43]. According to previous studies [19,20], however, earlier onsets and larger amplitudes of pre-landing EMG activity are considered important in preparing the foot positioning at touch-down. Sufficient pre-landing EMG activity is considered to be a crucial mechanism acting to protect the ligaments and joints from fall-related injury [24]. Since no significant differences in inversion kinematics were found between the two shoe conditions, the high-top shoe was not “rigid” enough to affect the ankle inversion. Meanwhile, our results showed that the high-top shoe partially delayed the pre-activation timing and decreased the amplitude of evertor muscle activity, which may decrease ankle joint stability and therefore increase the risk of ankle sprain. An alternative possibility is related to the participants’ perception of shoe collar height. High-top shoes may feel “safer” compared to low-top shoes, and this may subconsciously lead to later and lower muscle pre-activation, which may thus further be “deceived” into decreasing ankle stability. Such cognitive influences have been reported by Santello and McDonagh [44], who stated that participants relied on a continuous estimation of distance, time, or the environmental input to control EMG amplitude and muscle tension when they fell. Nevertheless, the above assumptions of how various collar heights may influence the neuromuscular response of the muscles deserve further investigation.

In the current study we didn’t occlude the vision of participants during the tests, for reasons of safety. This factor should be considered in the interpretation of the results, especially in the findings regarding pre-landing EMG activity. Vision is also a factor which may affect proper muscle pre-activity [45], but the interaction effects between shoe characteristics and visual control on leg muscle activation in landing have yet to be examined. Additionally, due to the experimental condition, ankle inversion has been measured in the current study using markers attached to the shoe, rather than by using markers attached directly to the foot or other possible methods such as dynamic x-ray imaging. Therefore, it should be noted that our observations may be subject to errors linked to deformation in the shoe or relative movement between shoe and foot.

## Conclusion

Shoe collar height did not influence the ankle inversion kinematics during landing on an inverted surface, yielding no changes in maximum ankle inversion angle, ankle inversion ROM, and maximum ankle inversion angular velocity. Therefore, the initial hypothesis was rejected as no decreased inversion was found with high-top shoes. However, the wearing of high-top shoes resulted in a significantly later onset time of the tibialis anterior and peroneus brevis muscle activity, and decreased pre-landing EMG activation of the ankle evertor muscles before contacting on the inverted (plus plantarflexed in some cases) surface. These findings provide preliminary evidence suggesting that a smaller muscular effort (a delayed pre-activation timing and decreased amplitude of evertor muscle activity) and changed proprioceptive feedback may result from wearing high-top shoes, and this might be detrimental to establishing and maintaining functional ankle joint stability in ankle strain situations.

## Abbreviations

3D: Three-dimensional; EMG: Electromyography; HS: High-top shoes; IEMG: Integrated EMG; LS: Low-top shoes; PB: Peroneus brevis; PL: Peroneus longus; ROM: Range of motion; TA: Tibialis anterior.

## Competing interests

WF, YF, YL, and JH have no competing interests to declare.

## Authors’ contributions

WF was the primary researcher involved in all aspects of the research and writing. YF provided data analysis, preparation of the manuscript. YL provided research study design, financial support, and critical review for this study and manuscript. JH provided data collection and processing. All authors read and approved the final manuscript. WF and YL contributed equally to the work.
